# The impact of the COVID-19 pandemic on psychiatric emergency consultations in adolescents

**DOI:** 10.1186/s40359-023-01085-7

**Published:** 2023-04-06

**Authors:** Pety So, André I. Wierdsma, Cornelis L. Mulder, Robert R. J. M. Vermeiren

**Affiliations:** 1 Youz, Center for Youth Mental Healthcare, Lupinestraat 1 2906 CV Capelle a/d Ijssel, Rotterdam, The Netherlands; 2 Parnassia Psychiatric Institute, Rotterdam, The Netherlands; 3 Epidemiological and Social Psychiatric Research Institute and Department of Psychiatry, Erasmus MC, University Medical Center, Rotterdam, The Netherlands; 4 Department of Child and Adolescent Psychiatry, Curium-LUMC, Leiden University Medical Center, Leiden, The Netherlands

**Keywords:** COVID-19, Adolescent mental health, Psychiatric emergency consultation, Externalizing disorders, Internalizing disorders

## Abstract

**Background:**

There is growing evidence that the COVID-19 pandemic, and its associated social distancing measures, affect adolescents’ mental health. We wanted to examine whether and how the number and characteristics of adolescents’ psychiatric emergency presentations have changed throughout the pandemic.

**Methods:**

We extracted data from the records of 977 psychiatric emergency consultations of adolescents aged 12- 19 who had been referred to the mobile psychiatric emergency services in Rotterdam, the Netherlands between January 1^st^ 2018 and January1^st^ 2022. Demographic, contextual, and clinical characteristics were recorded. Time-series-analyses were performed using quasi-Poisson Generalized Linear Model to examine the effect of the first and second COVID-19 lockdown on the number of psychiatric emergency consultations, and to explore differences between boys and girls and internalizing versus externalizing problems.

**Results:**

The number of psychiatric emergency consultations regarding adolescents increased over time: from about 13 per month in 2018 to about 29 per month in 2021. During the COVID-19 pandemic, the increase was tempered. In the second wave a pronounced increase of psychiatric emergencies among adolescents with internalizing problems but not with externalizing problems was found.

**Conclusion:**

Despite the reported increase of mental health problems in adolescents during the COVID-19 pandemic, we did find a smaller increase in psychiatric emergency consultations in this group then would be expected considering the overall trend. Besides changes in help-seeking and access to care, a possible explanation may be that a calmer, more orderly existence, or more parental supervision led to less psychiatric emergency situations in this age group. In the second wave the number of emergency consultations increased especially among girls with internalizing problems. While there has been a particular fall in emergency referrals of adolescents with externalizing problems since the start of the pandemic it is still too early to know whether this is a structural phenomenon. It would be important to elucidate whether the changes in emergency referrals reflect a true change in prevalence of urgent internalizing and externalizing problems in adolescents during the pandemic or a problem related to access to care.

## Introduction

The COVID-19 pandemic has proved to be a world-changing event with a major social and psychological impact on the global population. At the time of writing (early 2022), social-distancing measures such as the closing of schools and sports clubs were still substantially disrupting the lives of young people and their families, depriving adolescents of peer interactions and their daily routines [1–5]. When whole families are obliged to work at home, stress is likely to build up, especially in smaller living spaces.

As increasing evidence is now showing that adolescents’ mental health is being affected both by the pandemic and by the associated social-distancing measures [1, 4–8], it is relevant to establish whether the number and characteristics of adolescents’ psychiatric emergency presentations changed in the first 15 months of the pandemic, and, if so, how.

Pre-COVID-19 studies of emergency consultations involving young people showed that the number of mental health crises is higher during the months of school attendance than during school holidays [9–11]. The number is especially high after the summer break and at the end of the school year. High stress levels during school weeks can be attributed to the pressure to fit in with peers, to meet parents’ and teachers’ expectations, or to pass exams and get good grades [9]. The opening and closing of schools during the pandemic may thus have had a similar influence on the number of psychiatric emergency consultations involving adolescents. On the other hand, it is also true that schools serve as an important entry point for youth in need of help, and that home confinement may increase domestic conflicts, which in combination with pandemic related stress, may lead to an increased number of emergencies [12].

Although research on the impact of the COVID-19 pandemic on psychiatric emergencies in minors is limited, results indicate that the number of emergencies is reduced, but the presenting complaints are more serious. Two publications compared young people’s visits to emergency department for mental health (MH) reasons before and after the start of the COVID-19 pandemic [13, 14]. Unfortunately, neither study distinguished between the changes associated with the pandemic and the regular seasonal variations in emergency department visits. Using the medical records of all patients aged 5–24 who had presented at a US tertiary children’s hospital emergency department (ED), the first study [13] compared the period before the pandemic (from January 2018 to March 2020) with the first period of the restrictions (from April to December 2020). It found that the monthly mean number of ED visits for MH conditions decreased during the last nine months of 2020. However, a higher proportion of all ED visits involved MH conditions, and a higher proportion of the patients had needed hospitalization [13]. Another recent study [14], which was conducted in 10 different countries, also found that a lower total number of children and adolescents under age 19 presented with MH problems at hospital EDs during the first months of the COVID-19 lockdown. However, the proportion presenting with serious self-harm increased. In a large paediatric centre in France, the number of suicide attempts in children aged 15 years or younger showed a marked increase in late 2020 and early 2021 after the start of the pandemic [15]. Additionally, during the pandemic in the US in 2020 and early 2021, more ED visits were made by adolescents aged 12–17 years who had attempted suicide [16].

The increased admission rates and proportions of self-harm presentation found in these studies may indicate that help-seeking had been postponed [17].

As greater knowledge about such changes may help to clarify the pandemic’s short and long-term effects on adolescent mental health, we wished to increase our insight into the influences of the pandemic, social-distancing measures, and school closures on psychiatric emergency consultations of adolescents over time. We hypothesized that, early in the first school closure, stress levels were lower in the population aged 12–19, and health care system visits were postponed. This resulted in fewer psychiatric emergency consultations than in the same period in previous years. Regarding the second wave (starting October 2020), we expected to find that the number rose again, due partly to the accumulation of stress and fear, and partly to the fact that whole families had stayed indoors again. We were also interested to see whether there would be different patterns for boys and girls, and for internalizing problems and externalizing problems.

## Materials and methods

### Setting

The study was conducted in the Greater Rotterdam region (approximate population 1.2 million). Outpatient psychiatric emergency services throughout the Netherlands are responsible 24/7 for assessing patients of all ages (children, adolescents, and adults) with acute psychiatric problems referred to them. Their staff comprises community psychiatric nurses, physicians and psychiatrists, whose primary tasks are triage and the subsequent referral of psychiatric emergency patients to other psychiatric services.

During an emergency, patients are examined wherever they are by a team comprising a nurse and a physician or psychiatrist; if the physician is not a psychiatrist, a psychiatrist is consulted by telephone. If applicable, the team assesses not only the patient but also their significant others, and tries to resolve the crisis, preferably without hospitalization.

### Patients

Data for the period from January 1st, 2018 to January 1st, 2022 were extracted from the records of the mobile psychiatric emergency service. We included all 977 emergencies involving adolescents aged 12 to 19 who had been referred for urgent consultation.

The data was provided anonymously, and the authorized Medical Ethics Committee at Erasmus University Medical Center confirmed that the Medical Research Involving Human Subjects Act (known by its Dutch acronym, WMO) did not apply to this study, and thus that no informed consent was required (MEC-2020–0441).

### Data extraction

The study focused on the following variables:

Demographic characteristics, including age and sex.

Contextual characteristics: these included reasons for referral (defined as danger to self, danger to others, psychotic symptoms, depressive symptoms, and “other”); being in regular outpatient psychiatric care at time of the consultation (yes or no); and intervention (defined as no admission, voluntary admission, and compulsory admission).

Clinical characteristics: DSM 5 classifications and Global Assessment of Functioning (GAF) score. The DSM 5 classifications were grouped into internalizing disorders (e.g., depressive and anxiety disorders) and externalizing disorders (e.g., ADHD and behavioural disorders). If an individual patient was positive for both diagnostic categories, grouping was based on the reason for referral. Patients who combined internalizing and externalizing DSM 5 classifications and had been referred either because of depressive symptoms or because of a danger to themselves were grouped under internalizing disorders. Patients who had internalizing and externalizing DSM 5 classifications and had been referred with psychotic symptoms or a danger to others were grouped under externalizing disorders. As a total of 57 patients had no internalizing or externalizing DSM 5 classification, and the reason stated for their referral was ‘other’, they could not be grouped either under internalizing disorders or under externalizing disorders. These patients (2018 n = 8; 5.0%, 2019 n = 14; 6.2%, 2020 n = 12; 5.0%, 2021 n = 23; 6.6%) were left out in time-series analyses.

### Analyses

Descriptive statistics were used to summarize the characteristics of (1) all psychiatric emergency consultations; (2) the emergency consultations in the pre-COVID-19 period from January 2018 to March 2020; and (3) emergency consultations in the COVID-19 period from April 2020 to January 2022.

**Fig. 1:**
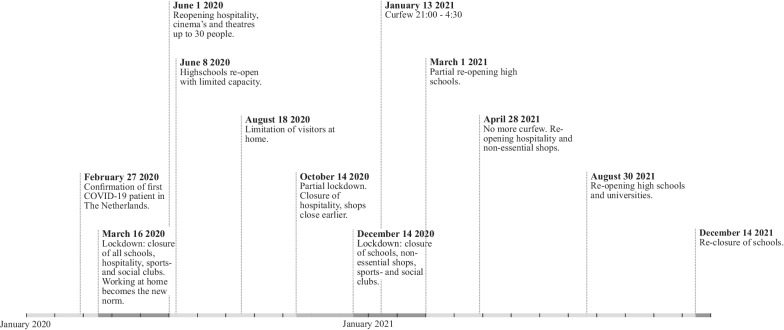
COVID restrictions in the Netherlands between January 1^st^, 2020 and January 1^st^, 2022

To model the time-series data, we used a quasi-Poisson Generalized Linear Models with a log link function and the number of emergency consultations in the previous month to account for autocorrelation [18]. Index of month and season indicator were used as fixed covariates to capture the overall trend and variability associated with school holidays. Effects of COVID-19 were modelled as indicator variables reflecting the first period of social-distancing measures, which in the Netherlands started in April 2020; and of the second period, which started in October 2020 [19]. For an overview of the COVID restrictions in the Netherlands in the period studied see Fig. 1.

Models including an interaction term of period and subgroups were tested to explore differences between sex and internalizing versus externalizing problems. Model selection was based on Wald-tests with alpha set at 10% to account for small effects due to low numbers of emergency consultations. All time-series analyses were conducted using the Statistical Software R (R Core Team, 2020).

## Results

In the four years study period, 977 psychiatric emergency assessments of adolescents aged 12–19 were performed by the psychiatric emergency service (median 31 (IQR19 – 41) referrals/month). Table 1 shows the characteristics for all consultations. A majority (60.9%) of the patients involved were girls; in 77.1% of all emergencies, patients had been referred due to risk of suicide or self-harm; and 21.5% had been admitted to a psychiatric hospital after consultation.

**Table 1:** Characteristics of all emergency consultations of adolescents aged 12–19 who had emergency consultations between January 1st, 2018 and January 1st 2022

	All	Pre-COVID-19*	COVID-19**
	n = 977	n = 443	n = 534
Girls n (%)	595 (60.9)	262 (59.1)	333 (62.4)
Mean age (SD)	16.2 (1.6)	16.2 (1.6)	16.1 (1.7)
Main reason for referral n (%)			
Risk of suicide/self-harm	753 (77.1)	326 (73.6)	427 (80.0)
Psychotic symptoms	98 (10.0)	43 ( 9.7)	55 (10.3)
Danger to others	51 ( 5.2)	28 ( 6.3)	23 ( 4.3)
Depressive symptoms	37 ( 3.8)	20 ( 4.5)	17 ( 3.2)
Other	38 ( 3.9)	26 ( 5.9)	12 ( 2.2)
In outpatient care n (%)	461 (47.2)	215 (48.5)	246 (46.1)
Internalizing problems n (%)	650 (66.5)	280 (63.2)	370 (69.3)
Externalizing problems n (%)	270 (27.6)	138 (31.2)	132 (24.7)
Other n (%)	57 ( 5.8)	25 ( 5.6)	32 ( 6.0)
Mean GAF score (SD)	39.7 (20.8)	38.3 (18.9)	40.8 (22.3)
Intervention n (%)			
Voluntary admission	129 (13.2)	59 (13.3)	70 (13.1)
Compulsory admission	81 ( 8.3)	40 ( 9.0)	41 ( 7.7)

*January 1st, 2018 to March 2020; ** April 2020 to January 2022; SD standard deviation; GAF global assessment of functioning.

The second and third columns of Table 1 contrast the characteristics of the patients who had had emergency consultation in the 27 months before the COVID-19 pandemic (median 17 (IQR 9–22) referrals/month), and in the first 21 months of the COVID-19 pandemic (median 40 (IQR 35–45) referrals/month). During the COVID-19 period, the proportion of externalizing problems was approximately 6 percent points lower than in the pre-COVID-19 period.

## Changes in the number of psychiatric emergency consultations

**Fig. 2:**
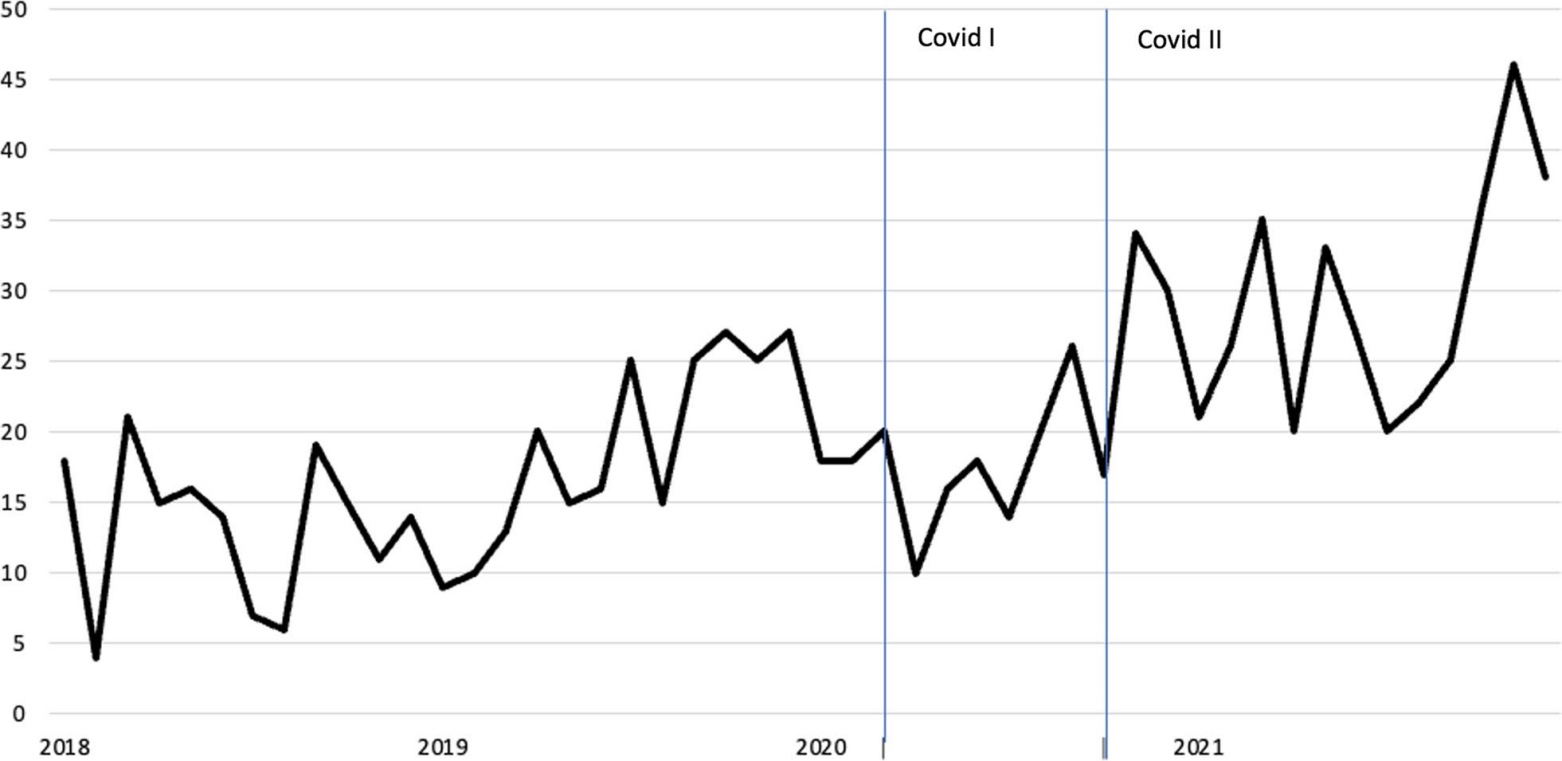
Number of psychiatric emergency consultations in adolescents aged 12–19 between January 1st, 2018 and January 1st, 2022. Covid I: March 2020—September 2020; Covid II: October 2020–December 2021

Figure 2 shows the number of psychiatric emergency consultations over the months studied (see Table 2 for model and model fit indices).

**Table 2:** Psychiatric emergency consultations: overall trend, seasonal effects, and COVID-19 effect

	B (SE)	p
Constant	2.311(0.129)	< 0.001
Lag Consults	– 0.005 (0.007)	0.511
Trend	0.032 (0.008)	< 0.001*
Season (ref. spring)		
Summer	– 0.238 (0.121)	0.058
Autumn	0.058 (0.116)	0.619
Winter	– 0.176 (0.128)	0.177
COVID-19	– 0.367 (0.182)	0.051

*p < 0.050; Goodness of Fit test: p =  < 0.001.

Overall, there is a trend towards an increase in the number of emergency consultations over time (B = 0.032, p =  < 0.001). Fewer adolescents are referred for emergency consultations in summer than in spring (B = -0.238, p = 0.058).

During the first 21 months of the COVID-19 pandemic, the number of psychiatric emergency consultations decreased, the decrease being more pronounced in the first months. As the pandemic progressed, the number of emergency consultations increased again, but the number of consultations still fell short of what might have been expected if the overall trend had continued (B = -0.367, p = 0.051).

## COVID-19 effects for boys and girls, and for internalizing problems and externalizing problems

**Fig. 3:**
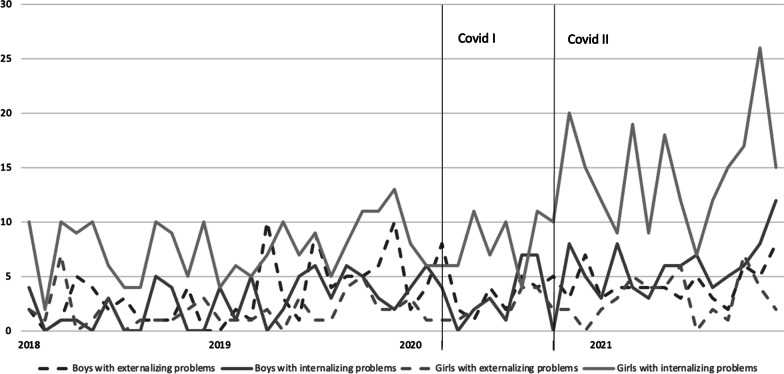
Number of psychiatric emergency consultations in adolescents aged 12–19 between January 1^st^, 2018 and January 1^st^ 2022 for the following: boys with externalizing problems, boys with internalizing problems, girls with externalizing problems, and girls with internalizing problems. Covid I: March 2020 – September 2020; Covid II: October 2020 – December 2021

Figure 3 shows the numbers of emergency consultations in the study period for the following groups: boys with externalizing problems, boys with internalizing problems, girls with externalizing problems and girls with internalizing problems (see Table 3 for model and model fit indices).

**Table 3:** Psychiatric emergency consultations: COVID-19 effects for boys and girls, and for internalizing problems and externalizing problems

	B (SE)	p
Constant	0.801 (0.170)	< 0.001
Lag Consults	– 0.014 (0.013)	0.293
Trend	0.031 (0.007)	< 0.001*
Patient group (ref. boys with externalizing problems)		
Boys with internalizing problems	– 0.217 (0.194)	0.265
Girls with externalizing problems	– 0.583 (0.216)	0.008 *
Girls with internalizing problems	0.894 (0.165)	< 0.001*
COVID-19 1	– 0.372 (0.289)	0.200
COVID-19 2	– 0.545 (0.272)	0.049 *
Interaction terms 1 (ref. boys with externalizing problems x COVID-19 1)		
Boys with internalizing problems x COVID-19 1	0.131 (0.391)	0.737
Girls with externalizing problems x COVID-19 1	0.073 (0.437)	0.867
Girls with internalizing problems x COVID-19 1	– 0.097 (0.325)	0.765
Interaction terms 2 (ref. boys with externalizing problems x COVID-19 2)		
Boys with internalizing problems x COVID-19 2	0.488 (0.277)	0.080
Girls with externalizing problems x COVID-19 2	0.164 (0.317)	0.607
Girls with internalizing problems x COVID-19 2	0.430 (0.241)	0.076

*p < 0.050; Goodness of Fit test: p =  < 0.001.

At the start of the COVID-19 pandemic, the number of consultations decreased in all groups. The increase in the second wave was more pronounced among boys and girls with internalizing problems than among boys with externalizing problems (B = 0.488, p = 0.080 and B = 0.430, p = 0.076).

## Discussion

This study describes the influence of the COVID-19 pandemic on the number of psychiatric emergencies in adolescent boys and girls with internalizing or externalizing problems until January 2022. We found an overall increase in the number of adolescent psychiatric emergency consultations over time, which rose from about 13 per months in 2018 to about 29 per month in 2021. During the COVID-19 pandemic the increase slowed. In the second wave, which in the Netherlands started in October 2020, the number of emergency consultations increased again, especially among girls with internalizing problems. Throughout the first and second COVID-19 waves, there were considerable monthly fluctuations in adolescents’ emergency presentations. However, despite the worldwide reported increase in mental health problems in adolescents during the pandemic [4–8], the number of psychiatric emergency consultations in this age group falls short of what one would expect if the overall trend had continued. No statements can be made whether these changes in emergency referrals reflect true changes in the prevalence of psychiatric emergencies in adolescents, or whether they are due to changes in help-seeking and access to care.

Our finding of a gradual increase in the number of emergency consultations which started before the pandemic is in line with other pre-COVID-19 studies that have reported an increase in psychiatric emergencies in adolescents [20–22]. Our finding of a decrease in psychiatric emergency consultations at the beginning of the pandemic – which may have resulted from postponed help-seeking – is also comparable with that in other studies [13–16].

Anxious reactions and the closure of schools, medical, and social services in the beginning of the pandemic, may have led to postponed help seeking and problems with access to care; if so, this may have delayed referrals for emergency consultation. Such a delay might partly explain the increase in of the number of emergency consultations during the second wave.

Another possible explanation for our finding of a reduction in crisis contacts during the first months of the pandemic is that there may have been a true reduction in urgent psychiatric problems in adolescents: the social-distancing measures and school closures may have led to a calmer and more orderly existence, which may in turn have led to less severe mental problems.

Our finding of a relative increase in consultations during the second wave is also in line with other studies [24–26]. The persistence of the COVID pandemic and the tightening of the restrictions may have led to exhaustion, especially in this age group, where sharing experiences with peers is crucial and autonomy is deeply sought [27]. Peer support can play a role in the prevention of internalizing problems by providing adolescents with a sense of belonging, practical assistance, emotional support, and reduce stress and negative emotions. School closure, unlimited Internet access, and boredom may have led to problematic internet usage, which is associated with mental health problems in adolescents [28, 29]. The fact that this increase in psychiatric emergencies was particularly marked in girls with internalizing problems adds to the findings of Yard et al. This study found an increase in adolescents with a suspected suicide attempt that was mainly due to consultations with adolescent girls who visited the ED during 2020 and early 2021 [17].

Our findings that, from the start of the pandemic, and during the second wave as well, there were fewer emergency consultations involving adolescents with externalizing problems is new. In literature, the impact of the pandemic on externalizing symptoms is not clear. Some studies found no changes in externalizing problems [30, 31] or substance abuse [32], others found pre-existing ADHD symptoms to worsen during the lockdown [^33^]. However, more parental supervision may have enabled some families to cope with adolescents with externalizing problems in the home situation, thereby reducing the number of emergency interventions needed.

### Strengths and limitations

The two main strengths of our study are the four years study period and the time-series analyses that allowed us to distinguish the changes associated with the pandemic from the regular trend.

The first of three main weaknesses concerns the possibility that the generalizability of our results was influenced by our small data set, and poor goodness of fit of the models. Important and potentially confounding variables like socioeconomic background were not available. The second weakness concerns the fact that, during psychiatric emergency consultations, clinicians based DSM 5 classifications on a clinical interview. However, since the division into internalizing and externalizing disorders was also based on the reason for referral, grouping will be broadly correct. Third, as the organization of psychiatric emergency health care for adolescents differs across regions and countries, care should be taken when generalizing our results.

## Conclusion

Despite the increase reported in mental health problems in adolescents during the COVID-19 pandemic, we found fewer emergency consultations in this group than one might expect based on the overall trend. While there has been a particular fall in emergency referrals of adolescents with externalizing problems since the start of the pandemic in April 2020 it is still too early to know whether this is a structural phenomenon. In the second wave a pronounced increase of psychiatric emergencies among adolescents with internalizing problems was found. It would be important to establish whether the changes in emergency referrals reflect a true change in the prevalence of urgent internalizing and externalizing problems in adolescents during the pandemic, or whether they are due to a problem related to access to care.

## Data Availability

The data that support the findings of this study are available from Aram van Reijsen (head of the mobile psychiatric emergency service, not one of the authors), but restrictions apply to the availability of these data, which were used under license for the current study, and so are not publicly available. Data are however available upon reasonable request and with the permission of Aram van Reijsen (Wijnhaven 100–110, 3011WN Rotterdam, The Netherlands, a.vanreijsen@anteszorg.nl).

## Abbreviations

DSM 5: Diagnostic and statistical manual of mental disorders, 5th Edition
ED: Emergency department
GAF: Global assessment of functioning
IQR: Interquartile range
MEC: Medical ethics committee
MH: Mental health
SD: Standard deviation
US: United states
WMO: Wet medisch-wetenschappelijk onderzoek met mensen
